# Intercorrelation between Immunological Biomarkers and Job Stress Indicators among Female Nurses: A 9-Month Longitudinal Study

**DOI:** 10.3389/fpubh.2014.00157

**Published:** 2014-10-13

**Authors:** Hyung-Suk Yoon, Kyoung-Mu Lee, Daehee Kang

**Affiliations:** ^1^Department of Biomedical Sciences, Seoul National University Graduate School, Seoul, South Korea; ^2^Department of Preventive Medicine, Seoul National University College of Medicine, Seoul, South Korea; ^3^Institute of Environmental Medicine, Seoul National University Medical Research Center, Seoul, South Korea; ^4^Department of Environmental Health, College of Natural Science, Korea National Open University, Seoul, South Korea; ^5^Cancer Research Institute, Seoul National University, Seoul, South Korea

**Keywords:** biomarker, immune, hydrocortisol, intercorrelation, job stress, nurse

## Abstract

Some immunological biomarkers have been reported to be associated with job-related stress. This study was conducted to explore the intercorrelation between the psychosocial components of job stress and various immunological biomarkers among female nurses. To assess monthly and weekly job stress, 41 nurses have repeatedly completed questionnaires such as the National Institute for Occupational Safety and Health General Job Stress Questionnaire, the profile of mood states short version and the Center for Epidemiologic Studies-Depression scale. Using flow cytometry and radioimmunoassay, the number of white blood cells, lymphocytic proliferation to mitogens, and toxoid were measured. Moreover, levels of hydrocortisol, interleukin-β, interferon-γ, and tumor necrosis factor-α and salivary immunoglobulin A were evaluated by enzyme-linked immunosorbent assay. When the Pearson correlation coefficients between job stress and immunological biomarkers were estimated after adjusting for age and smoking status, “Clashes: conflict at work” was significantly related to the number of CD4 cells (*r* = 0.36, *p*-value <0.05), CD4 to CD8 ratio (0.35; <0.05), response to concanavalin A (0.42; <0.05), and phytohemagglutinin (0.35; <0.05). Additionally, the level of hydrocortisol was significantly related to seven psychosocial measures; i.e., role conflict (−0.47; <0.01), role ambiguity (−0.39; <0.05), clashes at work (−0.38; <0.05), control and influence at work (0.53; <0.01), task control (0.55; <0.001), resources at work (0.35; <0.05), and skill underutilization (0.43; <0.05). The results indicate that (1) the psychosocial job stress is associated with the levels of some immunological biomarkers in nurses; and in particular, (2) hydrocortisol shows a remarkable relationship with diverse job stress indicators.

## Introduction

Stress is assumed to be a trigger that causes adverse conditions and/or deteriorates health. In the view of biological mechanism, stress is involved in immunological functions, which are linked with various diseases. In today’s modern society, there is an increase in common sources of stress in daily life, in particular, concerns about psychosocial pressures at workplace. Nemours studies have reported that job-related stress can affect individual susceptibility for diseases such as diabetes (1), hypertension (2), coronary heart disease (3), metabolic syndrome (4), and dementia (5).

Job stress may underlie the connection between immunological-inflammatory functions and human diseases. Studies demonstrated that job stressors could mediate the level of immunological biomarkers such as natural killer (NK) cells (6), suppressor T-cells (CD8)/NK cells (CD56)/interleukin (IL)-6 (7), CD4:CD8 ratio (7), and suppressor-inducer (CD4 + CD45RA) T lymphocytes (8). Furthermore, the alteration of the immune parameters was observed among some specific occupational groups prone to workplace stress such as blue-collar workers (9), power plant workers (10), university employees (11), white-collar workers (12, 13), and nurses (14–17). This indicates that job stress is assumed to act as a potential risk factor in cellular and/or humoral immune responses and induce immunological imbalance by itself.

In the previous study, we reported a significant variation in immunological biomarkers according to the high- vs. low-stress groups (17). Based on exploratory longitudinal study with repeated measures analysis, we found that nurses who experienced high levels of stress presented with lower levels of white blood cell (WBC) and tumor necrosis factor (TNF)-α, as well as higher levels of total salivary immunoglobulin A (sIgA) with statistical significance. Similarly, several studies were conducted to investigate the relationship between nurses’ job stress and immunological biomarkers (6, 7, 14–19). In observational studies exploring the job-related stress factors, nurses are considered to be one of the most appropriate subjects since (1) work-related stress level of nurses is relatively high; (2) job satisfaction is relatively lower than other occupational groups; and (3) recruitment of participants and data collection is more cooperative than other occupations. However, most of those studies focused on absolute levels of job stress but not for specific components of the stress constituting a total amount of job stress. Thus, the relationship between a specific factor of job stress and a single immunological biomarker was not fully understood.

Given the cause of job stress is multifactorial and heterogeneous, some specific job stress components such as job demand, role-conflicts, elimination in decision-making, high effort-low reward, and work shift may play an independent role in the stress-immune mechanism as an effect modifier. We hypothesized that some particular factors can be strongly correlated with immunological alterations and furthermore be a major contributor for health imbalance, identifying the specific contributor (i.e., the job stress components connected to immunological biomarkers) that may allow us to help prevent such negative health outcomes based on a tailored intervention that considers individuals’ job-related situation or working environment.

Along with the study hypothesis, the present study was conducted to investigate intercorrelations between specific components of psychosocial job stress and each immunological biomarker among female nurses. This qualitative approach will provide the evidence on how psychosocial job stress affects the human immune system; and identify the significant biomarker related to job stress in order to consider the causal mechanism involved in the stress-immune relationship.

## Materials and Methods

### Participants selection

The participants of this study were nurses who were volunteers recruited from a university hospital in the United States and were given written informed consent before entering the study. From a total of 1,043 nurses registered in the hospital, 514 nurses participated at the initiatory stage. Among these, subjects who were supposed to have any kinds of conditions that likely affected their immune system such as rheumatoid arthritis, thyroid disorders, cancer, systemic lupus erythematosus, myasthenia gravis, Graves’ disease, scleroderma, and any type of infection including human immunodeficiency virus (HIV) were excluded. Moreover, we strictly restricted the subjects to those who were highly likely to participate in the whole study for nine months. After an initial screening test and detailed questionnaire survey on health status, 41 premenopausal women were selected for the current study. The study protocol was reviewed and approved by the Johns Hopkins University School of Medicine and National Institute for Occupational Safety and Health (NIOSH). Detailed information has been described in previous paper (17).

### Data collection with questionnaires

During the subject’s work shift, a research assistant visited each subject and questionnaire surveys were conducted. Data were collected through face-to-face and self-administered questionnaires after blood and/or saliva collection. Demographic and work-related characteristics such as job title, work shift, and job continuity were investigated once during the first data collection. To assess specific components of psychosocial job stress, three types of questionnaires were used as below. Internal consistency of psychosocial variables (job stressors) was measured by the Cronbach’s alpha coefficient at the initial screening stage. Almost all psychosocial variables showed acceptable internal validity (Cronbach’s alpha coefficient ≥0.7).

#### Monthly questionnaire

The NIOSH General Job Stress Questionnaire (GJSQ) (20) was used to assess job stress of the study participants. This questionnaire was designed to measure a variety of job stressors and related-factors that may mediate the relationship between job stressors and health outcomes. Based on a total of nine repeated measurements (4 weeks apart from December to August) the full version of GJSQ was surveyed in the first and last month and the shorten version of GJSQ was administered rest of study period including job stressors and buffer factors. A list of the variables from the GJSQ used in the study is shown in Table 1. Regarding the interpretation of GJSQ, high-score means higher stress in job while high score on job control and buffer factors means low stress (20).

**Table 1:** **Summary statistics of monthly and weekly psychosocial variables**.

		Median	IQR
**MONTHLY PSYCHOSOCIAL VARIABLES FROM NIOSH GJSQ^a^**			
**Job stressor**	Clashes: conflict at work	3.11	2.72–3.62
	Control and influence at work	2.90	2.47–3.29
	Decision process control at work	2.63	2.38–3.29
	Group support: conflict at work	3.87	3.64–4.08
	Noncooperation between groups conflict	2.47	2.17–2.90
	Quantitative workload	3.59	3.43–3.94
	Resources at work control	2.39	1.93–3.00
	Responsibility for people	3.25	2.75–3.58
	Role ambiguity	2.40	1.98–3.13
	Role conflict	3.35	2.48–3.75
	Skill underutilization	3.89	3.07–4.33
	Task control at work	3.13	2.66–3.68
	Variance in workload	3.71	3.33–4.00
**Buffer factor**	Fellow workers social support	4.30	4.00–4.50
	Head nurse social support	3.46	3.14–4.25
	Spouse, friends, and family social support	4.72	4.29–4.85
**WEEKLY PSYCHOSOCIAL VARIABLE^b^**			
CES-D depression score		11.68	6.33–15.00
POMS anger subscale		3.33	1.06–4.31
POMS confusion subscale		2.87	1.42–4.20
POMS depression subscale		3.48	1.69–4.99
POMS frustration subscale		3.80	2.38–5.68
POMS tension subscale		4.07	2.71–6.23
POMS vigor subscale		15.29	11.87–17.62
Total POMS score		33.63	21.65–40.54

*IQR, interquartile range; CES-D, center for epidemiologic studies-depression; POMS, profile of mood status short form*.

*^a^Individual median and IQR values of the variables obtained from NIOSH (National Institute for Occupational Safety and Health) General Job Stress Questionnaire were calculated with the data from first month to ninth month*.

*^b^Individual median and IQR values were calculated with the data from 6th week to 33rd week*.

#### Weekly questionnaires

Job stress can interfere with negative mood changes including anxiety, tension, and fury and may further extend to moderate or severe depression. Given that our study subjects were all nurses whose job description has been generally reported to be associated with high rates of job stress, they may be more prone to mood disturbance and depression due to job-related stress. Thus, we conducted additional questionnaire surveys to evaluate the level of usual mood states and depression symptoms by using two types of questionnaires, i.e., Center for Epidemiologic Studies-Depression (CES-D) scale (21) and the profile of mood states (POMS) short form (22). The CES-D, a self-report scale used to estimate depressive symptoms, consists of 20 items representing the major components of depression and asks participants to rate how often they experienced depression symptoms over the past week. Ratings from 0 (rarely or none of the time) to 3 (most or all of the time) can be added up to 60, in which higher scores indicate more severe depressive states (21). The POMS short form consists of 37 mood related adjectives that are rated on a 0 (not at all) to 5 (extremely) scale. Six factor-based subscales, i.e., tension–anxiety, depression–dejection, anger–hostility, vigor–activity, fatigue–inertia, and confusion–bewilderment, were derived and total mood disturbance was estimated (22). Variables of the weekly questionnaire were presented in Table 1.

### Blood and saliva sampling

Under standardized protocol, blood (monthly collections), and saliva (weekly collections) samples were collected at least 1 h after meals from each of the participants. A phlebotomist congregated approximately 23 cc of blood from the antecubital site of each participant’s arms. Cells and serum were separated after centrifugation and lymphocytes and sera were evaluated with a panel of immunologic tests. Whole saliva samples were collected by using the spitting method. Accumulated saliva was expectorated into sterile 15 ml conical plastic tubes every 60 s for 5 min. The samples were stored at −80°C and shipped with dry ice in weekly batches to the NIOSH laboratory for analyses. All immunological measurements were done based on the rule of blind tests. Detailed sampling procedures were described in a previous study (16).

### Immunological biomarkers measurement

#### Natural killer cell cytolytic assay

An *in vitro* cytotoxicity assay using 51Cr-labeled Yac-1 cells was used. Splenocytes were adjusted to 1 × 10^7^ cells/ml in complete medium (RPMI, 10% fetal calf serum, 50 IU penicillin, and 50 μg streptomycin). After the 4-h incubation at 37°C and 5% CO_2_, 25 μl of supernatant was transferred to a 96-well plate containing solid scintillant. Plates were air dried overnight and after a 10-min dark delay on the Packard Top Count. The results are presented in lytic units per 10^7^ splenocytes using 10% lysis as the reference point.

#### Number of white blood cells

Number of white blood cells (per mm^3^) including T lymphocytes (CD3, CD4, CD8, CD4/CD8 ratio), B-lymphocytes (CD20), and NK cells (CD56) were assayed by immunofluorescence staining and flow-cytometry analysis.

#### Lymphocytic proliferation to mitogens or toxoid

Lymphocytic proliferation to mitogens (concanavalin A, phytohemagglutinin, and pokeweed) and toxoid (tetanus) were measured with a radioimmunoassay. Peripheral blood T lymphocyte cells were sorted by Ficoll-Paque density-gradients centrifugation, and washed with RPMI1640. [3H] TdR incorporated into cells was measured using a liquid scintillation counter (Packard, Meriden). The results were expressed as the average cpm. The delta-cpm was calculated as cpm incubated with mitogen or toxoid minus 3-day (phytohemagglutinin and concanavalin A) or 5-day (pokeweed mitogen and tetanus toxoid) control cpm.

#### Serum levels of cytokines; hydrocortisol, IL-1β, INF-γ, and TNF-α

Serum levels (ng/ml) of hydrocortisol, IL-1β, INF-γ, and TNF-α were assayed by enzyme-linked immunosorbent assay (ELISA) according to manufacturer’s manual (R&D Systems, Minneapolis, MN, USA).

#### Salivary IgA

Saliva samples were analyzed in a “blind” fashion for concentrations of total secretory IgA and end-point titers of specific sIgA against five combined strains of *E. coli* cell wall antigens (lipopolysaccharides, LPS) adjusted for total protein concentration (mg/ml), and salivary flow rate (ml/min). A modified ELISA method was used to measure both total and specific sIgA antibodies in the whole saliva samples. Detailed procedures were described in a previous study (16).

### Statistical analyses

Each of the study participants had up to nine data points for immunological measures of blood and monthly psychosocial variables (GJSQ) and up to 33 data points for immunological measures of saliva and weekly psychosocial variables (CES-D and POMS). Mean values of psychosocial variables and immunological biomarkers of each subject were calculated as arithmetic means during the study period. To identify our research hypotheses, the Pearson correlation coefficients between psychosocial variables and immunological biomarkers were estimated after adjusting for age and smoking status. All values of psychosocial variables and immunological biomarkers were log transformed for normal distribution. Furthermore, to avoid spurious association with false positive results due to extensive set of immunological biomarkers in multiple hypotheses testing, corrected *p* values were computed based on the statistical method for Benjamini–Hochberg false discovery rate (BH-FDR). Statistical analyses were performed with the SAS software version 9.2 (SAS Institute, Cary, NC, USA).

## Results

### Characteristics of study participants

The mean age was 29.9 years, 29% of the participants were smokers and about half were married, 38% of the participants were taking medicines at baseline, and 28% of participants were taking oral contraceptives. As for job characteristics, 57% of the participants were graduate nurses and clinical nurses, while others were clinical nurse masters and senior clinical nurses. Approximately 60% of the participants worked 8 or 12-h rotating shifts. They worked for an average of 39.1 h/week and had a 2.3-h overtime working per week. Job continuity at current job was an average of 4.6 years (Table 2).

**Table 2:** **Basic characteristics of study population**.

	Nurses (*N* = 41)
Age (year), mean ± SD	29.9 ± 5.9
Smoking status
No	27 (71.1)
Yes	11 (29.0)
Marital status
Married/living together	21 (55.3)
Single/never married	16 (42.1)
Single/divorced	1 (2.6)
Currently taking medicines at baseline
No	14 (37.8)
Yes	23 (62.2)
Oral contraceptive use
No	26 (72.2)
Yes	10 (27.8)
Family history of immune system disease
No	2 (5.3)
Yes	36 (94.7)
Job title
Graduate nurse/clinical nurse	21 (56.7)
Clinical nurse masters/senior clinical nurse	16 (43.3)
Work shift
Rotating 8/12 h	23 (60.5)
Permanent day/evening/night	6 (15.8)
Others	9 (23.7)
Hours worked per week, mean ± SD	39.1 ± 3.2
Hours worked overtime per week, mean ± SD	2.3 ± 2.5
Job continuity at current job (year), mean ± SD	4.6 ± 3.9

*Data are shown as number (%) or mean ± SD*.

### Summary statistics of psychosocial variables

Monthly psychosocial variables including job stressor and buffer factor were investigated by NIOSH GJSQ. The three highest variables (mean ± SD) among job stressors were conflict at work, 3.81 ± 0.57; quantitative workload, 3.71 ± 0.43; and variance in workload, 3.76 ± 0.59. Buffer factors were scored: fellow workers social support, 4.23 ± 0.44; head nurse social support, 3.64 ± 0.77; and spouse, friends, and family social support, 4.51 ± 0.51. The weekly psychosocial variable was quantified by POMS. The mean for the total POMS score was 33.1 ± 12.46 and CES-D depression score was 11.17 ± 6.23 (Median value and IQR of the psychosocial variables were shown in Table 1).

### Correlation between humoral immunological biomarkers and psychosocial variables

**Figure 1:**
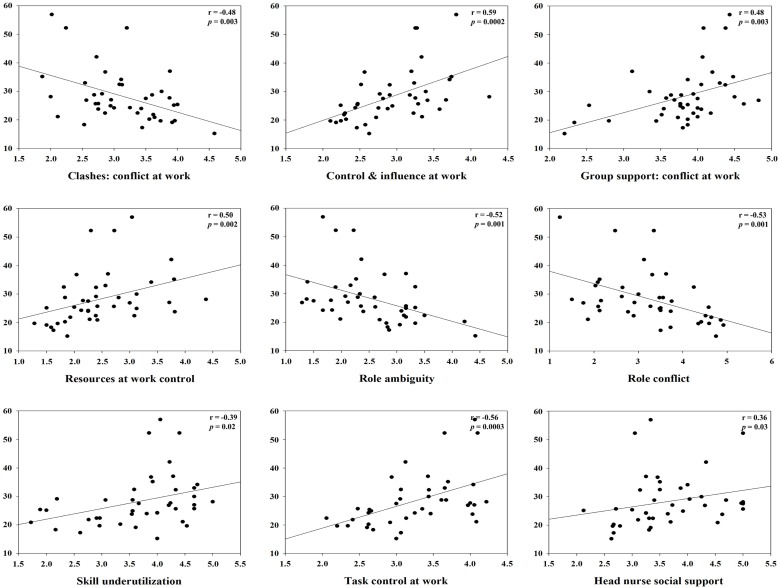
**Correlation between hydrocortisol and specific monthly job stress indicators with statistical significant (*p* < 0.05)**.

**Figure 2:**
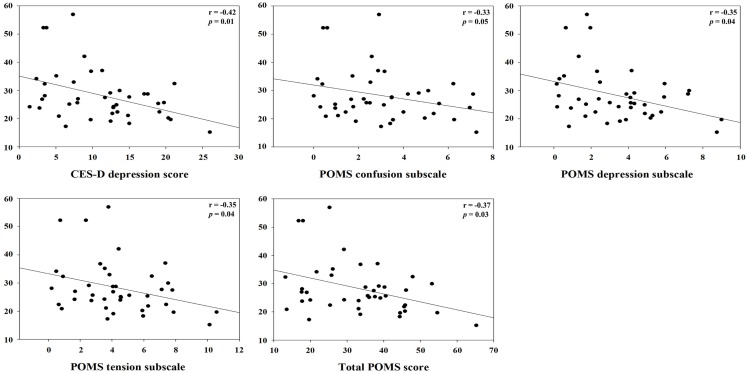
**Correlation between hydrocortisol and specific weekly job stress indicators with statistical significant (*p* < 0.05)**.

For clashes, conflict at work had a positive correlation with IL-1β (*r* = 0.45, *p* = 0.01) and specific sIgA (*r* = 0.34, *p* = 0.04), but a negative correlation with hydrocortisol (*r* = −0.48, *p* = 0.003). Control and influence at work had a positive correlation with hydrocortisol (*r* = 0.59, *p* = 0.0002). Decision process control at work had a positive correlation with hydrocortisol (*r* = 0.35, *p* = 0.04), but total sIgA had a negative correlation (*r* = −0.37, *p* = 0.03). For group support, conflict at work had a positive correlation with hydrocortisol (*r* = 0.48, *p* = 0.003) and INF-γ (*r* = 0.41, *p* = 0.01). Resources at work control had a positive correlation with hydrocortisol (*r* = 0.50, *p* = 0.002), but total sIgA had a negative correlation (*r* = −0.34, *p* = 0.04). Responsibility for people had a positive correlation with TNF-α (*r* = 0.42, *p* = 0.01). Role ambiguity had a positive correlation with specific sIgA (*r* = 0.38, *p* = 0.02), but hydrocortisol had a negative correlation (*r* = −0.52, *p* = 0.001). Role conflict had a negative correlation with hydrocortisol (*r* = −0.53, *p* = 0.001). Skill underutilization had a positive correlation with hydrocortisol (*r* = 0.39, *p* = 0.02) and TNF-α (*r* = 0.39, *p* = 0.02). Task control at work had a positive correlation with hydrocortisol (*r* = 0.56, *p* = 0.0003). The correlation between hydrocortisol and several job stressors, i.e., control and influence at work, resources at work control, role ambiguity, role conflict, and task control at work, remained statistically significant even after correcting for multiple comparisons (FDR <0.05). Hydrocortisol had a negative correlation with several weekly psychosocial variables such as CES-D depression (*r* = −0.42, *p* = 0.01), POMS depression subscale (*r* = −0.35, *p* = 0.04), POMS tension subscale (*r* = −0.35, *p* = 0.04), and total POMS score (*r* = −0.37, *p* = 0.03). Non-work last week had a positive correlation with specific sIgA (*r* = 0.36, *p* = 0.03). POMS vigor subscale had a negative correlation with INF-γ (*r* = −0.38, *p* = 0.02). Total POMS score had a negative correlation with INF-γ (*r* = −0.36, *p* = 0.03) (Table 3). The significant results are shown in Figures 1 and 2.

**Table 3:** **Pearson correlation coefficient^a^ (*r*, *p*-value) between monthly/weekly psychosocial variables and humoral immunological biomarkers**.

	Hydrocortisol	IL-β	INF-γ	TNF-α	Total sIgA	Specific sIgA
**MONTHLY PSYCHOSOCIAL VARIABLES FROM NIOSH GJSQ^b^**						
**Job stressor**
Clashes: conflict at work	−**0.48 (0.003)**	**0.45 (0.01)**	−0.20 (0.26)	−0.16 (0.35)	0.01 (0.97)	**0.34 (0.04)**
Control and influence at work	**0.59 (0.0002)^d^**	−0.03 (0.85)	0.17 (0.31)	0.21 (0.21)	−**0.33 (0.05)**	−0.08 (0.65)
Decision process control at work	**0.35 (0.04)**	−0.07 (0.69)	−0.02 (0.91)	0.27 (0.11)	−**0.37 (0.03)**	−0.05 (0.76)
Group support: conflict at work	**0.48 (0.003)**	−0.05 (0.78)	**0.41 (0.01)**	0.11 (0.52)	0.02 (0.92)	−0.28 (0.10)
Noncooperation between groups conflict	−0.15 (0.37)	0.10 (0.57)	−**0.33 (0.05)**	−0.16 (0.35)	−0.17 (0.32)	0.27 (0.11)
Quantitative workload	−0.19 (0.27)	−0.04 (0.84)	−0.17 (0.31)	−0.13 (0.47)	0.17 (0.33)	−0.21 (0.22)
Resources at work control	**0.50 (0.002)^d^**	−0.07 (0.69)	0.23 (0.19)	0.21 (0.22)	−**0.34 (0.04)**	−0.08 (0.65)
Responsibility for people	0.22 (0.20)	0.15 (0.39)	−**0.33 (0.05)**	**0.42 (0.01)**	−0.17 (0.33)	−0.05 (0.78)
Role ambiguity	−**0.52 (0.001)^d^**	−0.03 (0.88)	−0.26 (0.13)	0.09 (0.60)	−0.002 (0.99)	**0.38 (0.02)**
Role conflict	−**0.53 (0.001)^d^**	0.13 (0.45)	0.02 (0.92)	0.01 (0.95)	0.03 (0.86)	0.29 (0.09)
Skill underutilization	**0.39 (0.02)**	−0.02 (0.89)	−0.29 (0.09)	**0.39 (0.02)**	−0.03 (0.88)	−0.11 (0.52)
Task control at work	**0.56 (0.0003)^d^**	0.01 (0.96)	0.17 (0.34)	0.11 (0.53)	−0.22 (0.20)	−0.05 (0.77)
Variance in workload	−0.14 (0.43)	0.18 (0.28)	−0.24 (0.15)	−0.30 (0.08)	0.12 (0.48)	−0.002 (0.99)
**Buffer factor**
Fellow workers	**0.34 (0.04)**	0.05 (0.79)	0.01 (0.95)	−0.07 (0.70)	−0.20 (0.26)	−0.10 (0.57)
Head nurse	**0.36 (0.03)**	0.11 (0.51)	**0.39 (0.02)**	0.21 (0.23)	−0.19 (0.26)	−0.20 (0.24)
Spouse, friends, and family	0.28 (0.10)	−0.19 (0.26)	0.03 (0.87)	−0.001 (0.99)	−0.10 (0.56)	0.10 (0.55)
**WEEKLY PSYCHOSOCIAL VARIABLE^c^**						
CES-D depression score	−**0.42 (0.01)**	0.08 (0.63)	−0.14 (0.42)	0.16 (0.35)	0.03 (0.85)	0.22 (0.20)
POMS anger subscale	−0.32 (0.06)	0.32 (0.06)	−0.31 (0.07)	0.05 (0.78)	0.01 (0.95)	0.20 (0.24)
POMS confusion subscale	−**0.33 (0.05)**	0.12 (0.48)	−0.10 (0.56)	0.56 (0.80)	0.03 (0.88)	0.32 (0.06)
POMS depression subscale	−**0.35 (0.04)**	0.16 (0.37)	−0.20 (0.25)	0.25 (0.74)	−0.03 (0.88)	0.30 (0.08)
POMS frustration subscale	−0.32 (0.06)	0.08 (0.64)	−0.27 (0.12)	0.15 (0.39)	−0.32 (0.06)	**0.33 (0.05)**
POMS tension subscale	−**0.35 (0.04)**	0.15 (0.40)	−0.28 (0.10)	0.11 (0.52)	−0.003 (0.99)	0.22 (0.20)
POMS vigor subscale	−0.11 (0.52)	−0.18 (0.31)	−**0.38 (0.02)**	0.01 (0.97)	0.20 (0.26)	−0.04 (0.82)
Total POMS score	−**0.37 (0.03)**	0.14 (0.42)	−**0.36 (0.03)**	0.12 (0.48)	−0.01 (0.98)	0.27 (0.12)

*CES-D, center for epidemiologic studies-depression; POMS, profile of mood status short form; IL, interleukin; INF, interferon; TNF, tumor necrosis factor; sIgA, salivary immunoglobulin A. Bold font indicates significant values*.

*^a^Analyzed using Pearson partial correlations between variables after adjusting for age and smoking status*.

*^b^Individual mean values of the variables were calculated with the data from first month to ninth month*.

*^c^Individual mean values were calculated with the data from 6th week to 33rd week*.

*^d^BH-FDR adjusted *p* value was significant (*p* < 0.05)*.

### Correlation between cellular immunological biomarkers and psychosocial variables

Conflict at work had a positive correlation with helper T-cell (*r* = 0.36, *p* = 0.03), CD4/CD8 ratio (*r* = 0.40, *p* = 0.02), and concanavalin A (*r* = 0.43, *p* = 0.01). Decision process control had a negative association with the CD4/CD8 ratio (*r* = 0.37, *p* = 0.03). Quantitative workload had a positive correlation with T-cells (*r* = 0.38, *p* = 0.02), but it had a negative correlation with natural cell activity (*r* = −0.37, *p* = 0.03). Role ambiguity had a positive correlation with several cellular immune biomarkers, concanavalin A (*r* = 0.39, *p* = 0.02), phytohemagglutinin (*r* = 0.36, *p* = 0.03), and tetanus toxoid (*r* = 0.44, *p* = 0.01). Variance in workload had a negative correlation with natural cell activity (*r* = −0.37, *p* = 0.03) and NK cell (*r* = −0.37, *p* = 0.03). Natural cell activity (*r* = −0.35, *p* = 0.04) and NK cell (*r* = −0.34, *p* = 0.04) had a negative correlation with head nurse social support (Table 4).

**Table 4:** **Pearson correlation coefficient^a^ (*r*, *p*-value) between monthly/weekly psychosocial variables and cellular immunological biomarkers**.

	WBC	Lymphocytes	T cells (CD3)	Helper T-cell (CD4)	Suppressor T-cell (CD8)	CD4/CD8 ratio	B cell (CD20)	Natural killer cell (CD56)	Natural cell activity	Concanavalin A	Phytohemagglutinin	Pokeweed	Tetanus toxoid
**MONTHLY PSYCHOSOCIAL VARIABLES FROM NIOSH GJSQ^b^**													
**Job stressor**
Clashes: conflict at work	−0.08 (0.63)	0.24 (0.17)	0.28 (0.11)	**0.36 (0.03)**	−0.15(0.40)	**0.40 (0.02)**	−0.01 (0.98)	0.02 (0.91)	−0.10 (0.55)	**0.43 (0.01)**	0.33 (0.06)	0.10 (0.59)	0.004 (0.98)
Control and influence at work	−0.09 (0.62)	−0.19 (0.27)	−0.20 (0.25)	−0.23 (0.18)	0.001(0.99)	−0.19 (0.28)	0.04 (0.81)	−0.13 (0.45)	0.02 (0.91)	−0.05 (0.76)	−0.23 (0.18)	0.02 (0.91)	−0.15 (0.38)
Decision process control at work	0.13 (0.47)	−0.06 (0.72)	−0.11 (0.54)	−0.22 (0.21)	0.25(0.15)	−**0.37 (0.03)**	0.18 (0.31)	−0.08 (0.67)	0.04 (0.83)	−0.02 (0.92)	−0.30 (0.08)	−0.12 (0.50)	−0.20 (0.25)
Group support: conflict at work	0.06 (0.73)	−0.21 (0.24)	−0.21 (0.22)	−0.28 (0.11)	0.07(0.71)	−0.27 (0.12)	0.02 (0.93)	−0.12 (0.50)	−0.03 (0.87)	−0.26 (0.13)	−0.27 (0.12)	−0.08 (0.64)	−0.04 (0.83)
Noncooperation between groups conflict	−0.07 (0.70)	0.16 (0.36)	0.13 (0.47)	0.17 (0.34)	−0.04 (0.82)	0.16 (0.35)	0.06 (0.75)	0.19 (0.27)	0.18 (0.29)	0.21 (0.24)	0.20 (0.25)	0.02 (0.90)	0.16 (0.35)
Quantitative workload	0.12 (0.48)	0.31 (0.08)	**0.38 (0.02)**	0.29 (0.09)	0.26(0.14)	0.02 (0.93)	0.17 (0.33)	−0.30 (0.08)	−**0.37 (0.03)**	0.20 (0.25)	−0.18 (0.30)	−0.09 (0.63)	0.10 (0.56)
Resources at work control	0.08 (0.66)	−0.21 (0.24)	−0.19 (0.27)	−0.17 (0.34)	−0.13 (0.44)	−0.04 (0.84)	−0.02 (0.90)	−0.25 (0.15)	−0.10 (0.58)	−0.03 (0.86)	−0.23 (0.18)	0.03 (0.86)	−0.11 (0.53)
Responsibility for people	0.01 (0.50)	0.01 (0.97)	−0.07 (0.69)	0.02 (0.89)	−0.05 (0.77)	0.06 (0.74)	0.05 (0.79)	0.10 (0.57)	0.22 (0.19)	0.28 (0.10)	−0.26 (0.14)	0.06 (0.76)	0.13 (0.45)
Role ambiguity	0.20 (0.26)	0.22 (0.21)	0.20 (0.24)	0.21 (0.22)	0.05(0.79)	0.13 (0.45)	0.12 (0.50)	0.11 (0.55)	−0.18 (0.29)	**0.39 (0.02)**	**0.36 (0.03)**	0.13 (0.48)	**0.44 (0.01)**
Role conflict	0.15 (0.40)	0.30 (0.08)	**0.33 (0.05)**	**0.36 (0.03)**	−0.02(0.92)	0.29 (0.09)	0.09 (0.59)	−0.01 (0.95)	−0.21 (0.23)	0.33 (0.06)	0.23 (0.19)	0.07 (0.68)	0.27 (0.11)
Skill underutilization	−0.29 (0.10)	−0.11 (0.54)	−0.09 (0.63)	−0.05 (0.76)	−0.13(0.47)	0.05 (0.76)	−0.14 (0.41)	−0.22 (0.21)	−0.003 (0.99)	0.16 (0.35)	−0.23 (0.19)	0.26 (0.14)	0.21 (0.22)
Task control at work	−0.25 (0.16)	−0.16 (0.36)	−0.17 (0.34)	−0.19 (0.28)	−0.02 (0.89)	−0.13 (0.47)	0.001 (0.99)	−0.04 (0.80)	0.08 (0.64)	−0.07 (0.69)	−0.13 (0.45)	0.08 (0.67)	−0.11 (0.54)
Variance in workload	−0.29 (0.09)	0.04 (0.83)	0.15 (0.38)	0.10 (0.60)	0.06(0.75)	0.03 (0.88)	−0.04 (0.84)	−**0.37 (0.03)**	−**0.37 (0.03)**	0.17 (0.34)	−0.09 (0.61)	0.07 (0.70)	0.15 (0.39)
**Buffer factor**
Fellow workers	−0.09 (0.62)	−0.07 (0.68)	−0.08 (0.65)	−0.14 (0.41)	0.05(0.78)	−0.16 (0.37)	0.03 (0.89)	−0.09 (0.61)	−0.03 (0.87)	−0.20 (0.25)	−0.08 (0.66)	−0.20 (0.25)	−0.20 (0.25)
Head nurse	0.05 (0.78)	0.05 (0.80)	0.12 (0.49)	−0.02 (0.90)	0.22(0.20)	−0.20 (0.25)	−0.03 (0.87)	−**0.34 (0.04)**	−**0.35 (0.04)**	−0.05 (0.76)	−0.22 (0.21)	−0.12 (0.49)	−0.21 (0.23)
Spouse, friends, and family	−0.02 (0.92)	0.02 (0.90)	−0.01 (0.94)	−0.03 (0.87)	−0.02(0.93)	−0.02 (0.91)	**0.34 (0.05)**	0.06 (0.75)	−0.04 (0.82)	−0.07 (0.70)	−**0.38 (0.03)**	−0.17 (0.34)	0.15 (0.38)
**WEEKLY PSYCHOSOCIAL VARIABLE^c^**													
CES-D depression score	0.29 (0.09)	0.15 (0.38)	0.14 (0.43)	0.09 (0.61)	0.16 (0.35)	−0.06 (0.71)	0.0002 (0.99)	0.14 (0.43)	−0.07 (0.70)	0.24 (0.17)	0.06 (0.73)	−0.001 (0.99)	0.30 (0.08)
POMS anger subscale	0.14 (0.41)	0.13 (0.46)	0.19 (0.28)	0.07 (0.71)	0.23 (0.18)	−0.14 (0.43)	−0.14 (0.43)	−0.13 (0.46)	−0.18 (0.31)	0.11 (0.54)	−0.04 (0.81)	−0.13 (0.47)	−0.07 (0.67)
POMS confusion subscale	0.17 (0.34)	−0.08 (0.64)	−0.05 (0.80)	−0.07 (0.68)	−0.06 (0.75)	−0.01 (0.94)	−0.25 (0.15)	0.04 (0.83)	−0.14 (0.40)	0.22 (0.20)	0.18 (0.31)	0.09 (0.60)	0.23 (0.18)
POMS depression subscale	0.27 (0.13)	0.16 (0.36)	0.15 (0.39)	0.08 (0.64)	0.16 (0.36)	−0.07 (0.69)	−0.005 (0.98)	0.16 (0.36)	−0.10 (0.56)	0.27 (0.12)	0.02 (0.89)	−0.05 (0.77)	0.24 (0.16)
POMS frustration subscale	0.18 (0.32)	0.16 (0.37)	0.22 (0.20)	0.15 (0.40)	0.25 (0.15)	−0.09 (0.60)	−0.17 (0.34)	−0.10 (0.58)	−0.17 (0.32)	0.17 (0.34)	−**0.33 (0.05)**	−0.16 (0.38)	0.03 (0.87)
POMS tension subscale	0.10 (0.56)	0.03 (0.86)	0.11 (0.53)	0.07 (0.68)	−0.02 (0.91)	0.07 (0.70)	−0.27 (0.12)	−0.05 (0.80)	−0.09 (0.59)	0.21 (0.22)	0.10 (0.57)	0.10 (0.57)	0.14 (0.43)
POMS vigor subscale	0.08 (0.65)	0.23 (0.19)	0.26 (0.13)	0.20 (0.26)	**0.33 (0.06)**	−0.11 (0.53)	−0.07 (0.69)	0.03 (0.89)	−0.23 (0.18)	0.14 (0.44)	0.18 (0.23)	0.03 (0.86)	0.18 (0.30)
Total POMS score	0.13 (0.45)	0.13 (0.45)	0.21 (0.24)	0.13 (0.47)	0.21 (0.24)	−0.07 (0.69)	−0.24 (0.17)	−0.05 (0.76)	−0.22 (0.19)	0.19 (0.27)	−0.01 (0.97)	−0.02 (0.92)	0.12 (0.50)

*CES-D, center for epidemiologic studies-depression; POMS, profile of mood status short form; WBC, white blood cells. Bold font indicates significant values*.

*^a^Analyzed using Pearson partial correlation between variables after adjusting for age and smoking status*.

*^b^Individual mean values of the variables were calculated with the data from first month to ninth month*.

*^c^Individual mean values were calculated with the data from 6th week to 33rd week*.

## Discussion

This study was conducted to investigate the relationship between a specific component of psychosocial job stress and immunological biomarkers in female nurses. After correcting for multiple comparisons, the correlation between hydrocortisol and several job stressors, i.e., control and influence at work, resources at work control, role ambiguity, role conflict, and task control at work, remained with statistical significance. Though statistical significances were not secure throughout the multiple comparison corrections, several psychosocial job stressors presented the possibility that they could interfere with humoral and/or cellular immunological biomarkers.

Hydrocortisol (in body form, cortisol) appears to be a sensitive immunological biomarker for overall job stressors. Previous research expounded a phenomenon of cortisol secretion also known as the cortisol awakening response (CAR) (23) and levels of cortisol was influenced under physical conditions such as smoking (24), drinking (25), and physical activity (26). The present study focused on psychosocial conditions, and hydrocortisol was revealed to have positive correlations with several monthly immunological biomarkers such as control and influence at work, group support, skill underutilization, task control at work, and head nurse social support. Moreover, it was negatively correlated with clashes such as conflict at work, role ambiguity, role conflict, and several weekly psychosocial variables. Interestingly, the results exhibited consistency after controlling putative confounding factors such as taking oral contraceptives. Furthermore, several factors (control and influence at work, resources at work control, role ambiguity, role conflict, and task control at work) correlated with hydrocortisol remained statistically significant despite having been corrected for multiple comparisons. When people suffer from stress, the hypothalamic–pituitary–adrenal axis (HPA), which regulates the immunological response, is stimulated (27). The main functions of HPA are known to activate adrenocorticotropic hormone (ACTH) and glucocorticoid secretion. The activation of HPA induces the adrenal cortex stimulation and zona fasciculate secretion of cortisol, a glucocorticoid hormone, which plays a role in physiological function during stressful circumstances (27). This indicates that cortisol could be directly induced during the initiation of stress in anti-stress pathways, and our findings support the biological connections between cortisol and stress (i.e., work place related stress). In the same vein, studies have also reported the association between cortisol and job stress although the findings were inconsistent (1) cortisol could decrease as a result of high-job strain among female health care providers because stress caused circadian rhythm disturbance (28); (2) cortisol level was higher in participants with low-job strain than that of the other participants (29); (3) job demand was negatively correlated with cortisol (30); but (4) a study on assembly line workers reported that cortisol had a positive correlation with some self-report psychosocial variables such as irritation, pressed by demands, time pressure, and tired during work days (31). The inconsistencies may be explained by the variations in design methodology and workplace circumstances. Thus, further studies are needed to investigate the relationship of various job stressors on hydrocortisol alterations and to elucidate the causal mechanisms involved in job-related stress and immune responses.

Despite inconsistent with previous findings between psychometric parameters and humoral immunological biomarkers such as IL-1β, INF-γ, and TNF-α, our results showed that IL-1β, INF-γ, TNF-α, total sIgA, and specific sIgA may be significantly correlated with various job stress indicators. TNF-α showed a positive correlation with responsibility for people and skill underutilization. But in general, decreased TNF-α meant activation of humoral immune function during stress (17, 32). INF-γ, which plays a similar role like TNF-α, had irregular correlations with both positive correlations in group support (conflict at work and head nurse social support) and negative correlation with non-cooperation between group conflicts and responsibility for people in our study, whereas a previous study indicated that home-related social support may affect the level of INF-γ (33). These inconsistencies may be induced due to the differences in the study population and/or methodology or insufficient statistical power of the present study. Replication studies with a greater number of study participants should be conducted to clarify the ambiguity.

No obvious associations were found between cellular immunological biomarkers and job stress, whereas cellular immunological biomarkers including T-cells (CD3), helper T-cell (CD4), NK cell (CD56), and NK cell activity showed a putative relationship with several specific job stress indicators. Concretely, T-cells (CD3) increasingly reacted when quantitative workload or role conflict was high. Moreover, both clashes, conflict at work and role conflict, had significant influence on helper T-cells (CD4). Previous studies have reported inconsistent results between T lymphocytes and job stress. The level of T-cells (CD3) was significantly lower in the higher strain group (8), and job control had a positive correlation with a subpopulation of helper T-cells, not helper T-cell (CD4) (9). Nakata and colleagues partly explained the differences in that (1) potential confounders were not fully considered such as alcohol drinking and physical activity, and the possibility of (2) a small sample size (8). T-cell (CD3) was also reported to be indirectly affected by social support (33).

Natural killer cell (CD56) appears to be negatively correlated with variance in workload and head nurse social support. It also has been reported that job satisfaction shows a dose-dependent relationship (12) and a negative correlation with quantitative workload and head nurse social support. Previous studies support our results that job stress leads to decreased NK cell (CD56) (14) and NK cell activity (6) in female nurses, and NK cell activity also had a negative correlation with quantitative workload (34). Moreover, Miyazaki and colleagues reported that high-social support was directly related to NK cell levels (33). On the contrary, it is generally known that (1) job stress could be reduced by social support (35); (2) high supervisory support could decrease job stress in nurse aids (36); and (3) lower log salivary IgA levels was reveled in teachers without supervisory support (37), but our results showed that head nurse (supervisor) social support caused low-immune function by NK cell (CD56) and NK cell activity. These conflicting findings are assumed to be due to bilaterality in social supports; thus, a systematic approach to the immunological harmful effects of excessive support from a supervisor is needed in future studies.

In our previous study, the total number of WBC significantly decreased among the high-objective stress group (17), whereas this was not replicated in the present study. In terms of the correlation between WBC and job stress indicators, compatible results have been reported (34); therefore, further examinations are still needed. The CD4:CD8 ratio also did not show any significant association in this study, but low reward and/or high effort–reward imbalance has been reported to affect the variation in the CD4:CD8 ratio (7). These inconsistencies may be derived from the differences between specific job stress indicators and overall rating stress, and thus, further studies will consider more detailed groupings and analyses regarding the questionnaire data.

Several study limitations should be noted. First, since the present study was conducted with a small number of study participants, we did not secure the sufficiency of the statistical power. The restricted sample size may have yielded null results and/or may not have detected the hidden association underlying the biological pathway. Furthermore, this did not allow stratified analyses according to putative covariates known to be involved with the job stress mechanism. However, our preliminary findings provide clues for further studies on repeated measures and multiple immunological biomarkers and job stress indicators including daily mood and depressive symptoms. Second, potential bias such as measurement errors and selection bias may not have been completely eliminated. Considering all psychosocial variables were based on self-reported data, the possibility of response bias may also exist. Finally, our participants could not fully represent the normal population due to the convenient sampling method and the limited number of participants. To generalize the findings of the present study, further replication studies covering the general population with sufficient sample size are warranted. Thus, any interpretation should be done cautiously. Despite the limitations, our longitudinal study design could provide more integrated results, with its periodic data collection, various job stress questionnaires, larger number of assays, and multiple biomarkers. Further studies will assure better understanding of the hidden relationships between job stress and immunological biomarkers.

Our findings suggest that several specific components of psychosocial job stress are significantly associated with some immunological biomarkers. Hydrocortisol particularly showed a remarkable relationship with diverse job stress indicators (control and influence at work, resources at work control, role ambiguity, role conflict, and task control at work) after multiple comparison tests and its biological plausibility was also supported. Given that specific circumstances at workplaces can intimately interfere with immunological alterations and be a major contributor for health imbalance, our findings can aid in setting up tailored intervention by taking into consideration individuals’ job-related situation or working environment, and further help to prevent adverse health consequences involved in the stress-immune relationship.

## Conflict of Interest Statement

The authors declare that the research was conducted in the absence of any commercial or financial relationships that could be construed as a potential conflict of interest.

## Supplementary Material

The Supplementary Material for this article can be found online at http://www.frontiersin.org/Journal/10.3389/fpubh.2014.00157/abstract

## References

[1] Heraclides A Chandola T Witte DR Brunner EJ Psychosocial stress at work doubles the risk of type 2 diabetes in middle-aged women: evidence from the Whitehall II study. Diabetes Care (2009) 32:2230–5 10.2337/dc09-0132 19720842 PMC2782982

[2] Spruill TM Chronic psychosocial stress and hypertension. Curr Hypertens Rep (2010) 12:10–6 10.1007/s11906-009-0084-8 20425153 PMC3694268

[3] Aboa-Eboulé C Brisson C Maunsell E Mâsse B Bourbonnais R Vézina M Job strain and risk of acute recurrent coronary heart disease events. JAMA (2007) 298:1652–60 10.1001/jama.298.14.1652 17925517

[4] Chandola T Brunner E Marmot M Chronic stress at work and the metabolic syndrome: prospective study. BMJ (2006) 332:521–5 10.1136/bmj.38693.435301.80 16428252 PMC1388129

[5] McNeely E The consequences of job stress for nurses’ health. Time for a check-up. Nurs Outlook (2005) 53:291–9 10.1016/j.outlook.2005.10.001 16360700

[6] Morikawa Y Kitaoka-Higashiguchi K Tanimoto C Hayashi M Oketani R Miura K A cross-sectional study on the relationship of job stress with natural killer cell activity and natural killer cell subsets among healthy nurses. J Occup Health (2005) 47:378–83 10.1539/joh.47.378 16230830

[7] Amati M Tomasetti M Ciuccarelli M Mariotti L Tarquini LM Bracci M Relationship of job satisfaction, psychosocial distress and stress-related biological parameters among healthy nurses. A longitudinal study. J Occup Health (2010) 52:31–8 10.1539/joh.L9042 20032591

[8] Nakata A Araki S Tanigawa T Miki A Sakurai S Kawakami N Decrease of suppressor-inducer (CD4+CD45RA) T lymphocytes and increase of serum immunoglobulin G due to perceived job stress in Japanese nuclear electric power plant workers. J Occup Environ Med (2000) 42(2):143–50 10.1097/00043764-200002000-00007 10693074

[9] Kawakami N Tanigawa T Araki S Nakata A Sakurai S Yokoyama K Effects of job strain on helper-inducer (CD4+CD29+) and suppressor-inducer (CD4+CD45RA+) T cells in Japanese blue-collar workers. Psychother Psychosom (1997) 66:192–8 10.1159/000289134 9259042

[10] Nakata A Tanigawa T Fujioka Y Kitamura F Iso H Shimamoto T Association of low job control with a decrease in memory (CD4+CD45RO+) T lymphocytes in Japanese middle-aged male workers in an electric power plant. Ind Health (2002) 40:142–8 10.2486/indhealth.40.142 12064555

[11] Boscolo P Di Donato A Di Giampaolo L Forcella L Reale M Dadorante V Blood natural killer activity is reduced in men with occupational stress and job insecurity working in a university. Int Arch Occup Environ Health (2009) 82:787–94 10.1007/s00420-008-0374-5 18941771

[12] Nakata A Takahashi M Irie M Swanson NG Job satisfaction is associated with elevated natural killer cell immunity among healthy white-collar employees. Brain Behav Immun (2010) 24:1268–75 10.1016/j.bbi.2010.05.004 20561922

[13] Nakata A Takahashi M Irie M Effort-reward imbalance, overcommitment, and cellular immune measures among white-collar employees. Biol Psychol (2011) 88:270–9 10.1016/j.biopsycho.2011.08.012 21889570

[14] De Gucht V Fischler B Demanet C Immune dysfunction associated with chronic professional stress in nurses. Psychiatry Res (1999) 85:105–11 10.1016/S0165-1781(98)00131-0 10195321

[15] Endresen IM Vaernes R Ursin H Tonder O Psychosocial stress-factors and concentration of immunoglobulins and complement components in Norwegian nurses. Work Stress (1987) 1:365–75 10.1080/02678378708258527

[16] Henningsen GM Hurrell JJ Jr Baker F Douglas C MacKenzie BA Robertson SK Measurement of salivary immunoglobulin A as an immunologic biomarker of job stress. Scand J Work Environ Health (1992) 18:133–6 1514073

[17] Lee KM Kang D Yoon K Kim SY Kim H Yoon HS A pilot study on the association between job stress and repeated measures of immunological biomarkers in female nurses. Int Arch Occup Environ Health (2010) 83:779–89 10.1007/s00420-010-0544-0 20496079

[18] Copertaro A Bracci M Gesuita R Carle F Amati M Baldassari M Influence of shift-work on selected immune variables in nurses. Ind Health (2011) 49:597–604 10.2486/indhealth.MS1210 21804267

[19] Fukuda H Ichinose T Kusama T Yoshidome A Anndow K Akiyoshi N The relationship between job stress and urinary cytokines in healthy nurses: a cross-sectional study. Biol Res Nurs (2008) 10:183–91 10.1177/1099800408323219 18829600

[20] Hurrell JJ Jr McLaney MA Exposure to job stress – a new psychometric instrument. Scand J Work Environ Health (1988) 14(Suppl 1):27–8 3393871

[21] Radloff LS The CES-D scale a self-report depression scale for research in the general population. Appl Psychol Meas (1977) 1:385–401 10.1177/014662167700100306

[22] Shacham S A shortened version of the profile of mood states. J Pers Assess (1983) 47:305–6 10.1207/s15327752jpa4703_14 6886962

[23] Fries E Dettenborn L Kirschbaum C The cortisol awakening response (CAR): facts and future directions. Int J Psychophysiol (2009) 72:67–73 10.1016/j.ijpsycho.2008.03.014 18854200

[24] Rohleder N Kirschbaum C The hypothalamic-pituitary-adrenal (HPA) axis in habitual smokers. Int J Psychophysiol (2006) 59:236–43 10.1016/j.ijpsycho.2005.10.012 16325948

[25] Badrick E Bobak M Britton A Kirschbaum C Marmot M Kumari M The relationship between alcohol consumption and cortisol secretion in an aging cohort. J Clin Endocrinol Metab (2008) 93:750–7 10.1210/jc.2007-0737 18073316 PMC2266962

[26] Salmon P Effects of physical exercise on anxiety, depression, and sensitivity to stress. A unifying theory. Clin Psychol Rev (2001) 21:33–61 10.1016/S0272-7358(99)00032-X 11148895

[27] Arafah BM Hypothalamic pituitary adrenal function during critical illness: limitations of current assessment methods. J Clin Endocrinol Metab (2006) 91:3725–45 10.1210/jc.2006-0674 16882746

[28] Fujiwara K Tsukishima E Kasai S Masuchi A Tsutsumi A Kawakami N Urinary catecholamines and salivary cortisol on workdays and days off in relation to job strain among female health care providers. Scand J Work Environ Health (2004) 30:129–38 10.5271/sjweh.770 15127783

[29] Steptoe A Wardle J Lipsey Z Mills R Oliver G Jarvis M A longitudinal study of work load and variations in psychosocial well-being, cortisol, smoking, and alcohol consumption. Ann Behav Med (1998) 20:84–91 10.1007/BF02884453 9989313

[30] Sluiter JK Frings-Dresen MH van der Beek AJ Meijman TF Heisterkamp SH Neuroendocrine reactivity and recovery from work with different physical and mental demands. Scand J Work Environ Health (2000) 26:306–16 10.1136/oem.57.5.298 10994796

[31] Lundberg U Granqvist M Hansson T Magnusson M Wallin L Psychosocial and physiological stress responses during repetitive work at an assembly line. Work Stress (1989) 3:143–53 10.1080/02678378908256940

[32] Marshall GD Jr Agarwal SK Lloyd C Cohen L Henninger EM Morris GJ Cytokine dysregulation associated with exam stress in healthy medical students. Brain Behav Immun (1998) 12:297–307 10.1006/brbi.1998.0537 10080859

[33] Miyazaki T Ishikawa T Nakata A Sakurai T Miki A Fujita O Association between perceived social support and Th1 dominance. Biol Psychol (2005) 70:30–7 10.1016/j.biopsycho.2004.09.004 15979778

[34] Kawaguchi Y Toyomasu K Yoshida N Baba K Uemoto M Minota S Measuring job stress among hospital nurses: an attempt to identify biological markers. Fukuoka Igaku Zasshi (2007) 98:48–55 17396571

[35] AbuAlRub RF Job stress, job performance, and social support among hospital nurses. J Nurs Scholarsh (2004) 36:73–8 10.1111/j.1547-5069.2004.04016.x 15098422

[36] McGilton KS Hall LM Wodchis WP Petroz U Supervisory support, job stress, and job satisfaction among long-term care nursing staff. J Nurs Adm (2007) 37:366–72 10.1097/01.NNA.0000285115.60689.4b 17939468

[37] Masilamani R Darus A Ting AS Ali R Mahmud AB David K Salivary biomarkers of stress among teachers in an urban set. Asia Pac J Public Health (2012) 24:278–87 10.1177/1010539510393725 21385771

