# Supplementary material for: Intercorrelation between Immunological Biomarkers and Job Stress Indicators among Female Nurses: A 9-Month Longitudinal Study
Source: Front Public Health. 2014 Oct 13;2:157. doi: 10.3389/fpubh.2014.00157 (PMC4195281; doi:10.3389/fpubh.2014.00157)
Supplement: Supplementary file 1 [file Data_Sheet_1.PDF]

Appendix1. **Pearson correlation coefficient [a] (r, *p*-value) between monthly/weekly psychosocial variables and humoral immunological biomarkers: Results without log-transformation**

	Hydrocortisol	IL-β	INF-γ	TNF-α	Total sIgA	Specific sIgA
**Monthly psychosocial variables from NIOSH GJSQ [a]**						
**Job stressor**						
Clashes: conflict at work	**-0.48 (0.003)**	**0.35 (0.03)**	-0.14 (0.44)	-0.21 (0.21)	0.08 (0.64)	**0.30 (0.05)**
Control & influence at work	**0.56 (0.0004)**	-0.03 (0.88)	0.04 (0.84)	0.20 (0.24)	**-0.32 (0.05)**	-0.02 (0.93)
Decision process control at work	0.35 (0.04)	-0.01 (0.95)	-0.13 (0.47)	0.27 (0.11)	**-0.32 (0.05)**	-0.04 (0.81)
Group support: conflict at work	**0.43 (0.009)**	-0.01 (0.96)	**0.29 (0.09)**	0.10 (0.57)	0.07 (0.67)	-0.33 (0.06)
Noncooperation between groups conflict	-0.16 (0.37)	0.16 (0.34)	-0.21 (0.22)	-0.20 (0.25)	-0.11 (0.53)	0.25 (0.14)
Quantitative workload	-0.20 (0.27)	-0.14 (0.41)	-0.13 (0.46)	-0.13 (0.45)	0.28 (0.10)	-0.14 (0.43)
Resources at work control	**0.41 (0.02)**	-0.06 (0.71)	0.11 (0.52)	0.20 (0.24)	**-0.30 (0.07)**	-0.10 (0.57)
Responsibility for people	0.20 (0.25)	0.06 (0.71)	**-0.40 (0.02)**	**0.33 (0.04)**	-0.08 (0.64)	-0.11 (0.51)
Role ambiguity	**-0.51 (0.002)**	-0.09 (0.61)	-0.24 (0.17)	0.10 (0.54)	-0.03 (0.86)	**0.30 (0.07)**
Role conflict	**-0.53 (0.001)**	0.13 (0.45)	0.05 (0.79)	0.03 (0.85)	0.08 (0.63)	0.23 (0.19)
Skill underutilization	**0.39 (0.02)**	-0.06 (0.74)	**-0.36 (0.03)**	**0.29 (0.09)**	-0.01 (0.96)	-0.001 (0.99)
Task control at work	**0.56 (0.0004)**	0.08 (0.62)	0.05 (0.77)	0.10 (0.55)	-0.23 (0.18)	-0.01 (0.94)
Variance in workload	-0.15 (0.38)	-0.01 (0.96)	-0.20 (0.25)	-0.29 (0.08)	0.27 (0.11)	-0.06 (0.73)
**Buffer factor**						
Fellow workers	**0.34 (0.04)**	0.13 (0.44)	0.01 (0.96)	-0.07 (0.70)	-0.26 (0.13)	-0.13 (0.46)
Head nurse	**0.29 (0.09)**	0.14 (0.42)	0.24 (0.16)	0.20 (0.26)	-0.19 (0.26)	-0.05 (0.77)
Spouse, friends & family	0.28 (0.11)	-0.22 (0.20)	0.04 (0.83)	-0.01 (0.97)	-0.07 (0.67)	0.11 (0.52)
**Weekly psychosocial variable [b]**						
CES-D depression score	**-0.45 (0.01)**	0.13 (0.46)	-0.18 (0.28)	0.21 (0.22)	0.01 (0.95)	0.26 (0.12)
POMS anger subscale	**-0.46 (0.01)**	0.26 (0.12)	-0.27 (0.12)	0.11 (0.54)	0.04 (0.80)	0.22 (0.19)
POMS confusion subscale	**-0.32 (0.05)**	0.13 (0.44)	-0.11 (0.51)	0.09 (0.61)	0.03 (0.88)	**0.33 (0.05)**
POMS depression subscale	**-0.39 (0.02)**	0.13 (0.45)	-0.18 (0.29)	0.17 (0.33)	-0.01 (0.85)	**0.40 (0.02)**
POMS frustration subscale	-0.32 (0.06)	0.05 (0.79)	-0.21 (0.23)	0.17 (0.31)	-0.30 (0.08)	**0.47 (0.01)**
POMS tension subscale	**-0.42 (0.01)**	0.24 (0.17)	**-0.37 (0.03)**	0.11 (0.51)	-0.002 (0.99)	**0.33 (0.05)**
POMS vigor subscale	-0.17 (0.31)	-0.14 (0.41)	**-0.39 (0.02)**	0.04 (0.82)	0.21 (0.22)	-0.08 (0.64)
Total POMS score	**-0.42 (0.01)**	0.10 (0.56)	**-0.36 (0.03)**	0.14 (0.41)	-0.01 (0.95)	0.32 (0.06)

Abbreviation: CES-D, Center for Epidemiologic Studies-Depression; POMS, Profile of Mood Status Short Form; IL, interleukin; INF, interferon; TNF, tumor necrosis factor; sIgA, secretory immunoglobulin A;

a. Analyzed using Pearson partial correlations between variables after adjusting for age and smoking status
b. Individual mean values of the variables obtained from NIOSH (National Institute for Occupational Safety and Health) General Job Stress Questionnaire were calculated with the data from 1st month to 9th month
c. Individual mean values were calculated with the data from 6th week to 33rd week

Appendix2. Pearson correlation coefficient [a] (r, *p*-value) between monthly/weekly psychosocial variables and cellular immunological biomarkers: Results without log-transformation

	WBC	Lympho -cytes	T cells (CD3)	Helper T-cell (CD4)	Suppressor T-cell (CD8)	CD4/CD8 ratio	B cell (CD20)	Natural killer cell (CD56)	Natural cell activity	Concana -valin A	Phyto- heama- gglutinin	Pokeweed	Tentanus toxoid
**Monthly psychosocial variables from NIOSH GJSQ [b]**													
**Job stressor**													
Clashes: conflict at work	-0.07 (0.70)	0.26 (0.14)	0.29 (0.09)	**0.38 (0.03)**	-0.15 (0.39)	**0.36 (0.03)**	-0.01 (0.96)	0.02 (0.92)	-0.25 (0.15)	**0.44 (0.01)**	**0.35 (0.04)**	0.14 (0.42)	-0.05 (0.77)
Control & influence at work	-0.11 (0.54)	-0.20 (0.25)	-0.19 (0.27)	-0.24 (0.18)	0.02 (0.90)	-0.14 (0.42)	0.01 (0.93)	-0.06 (0.75)	0.24 (0.17)	-0.02 (0.93)	-0.29 (0.09)	0.01 (0.98)	-0.10 (0.57)
Decision process control at work	0.10 (0.56)	-0.09 (0.61)	-0.11 (0.54)	-0.23 (0.18)	0.31 (0.07)	**-0.30 (0.08)**	0.13 (0.46)	0.03 (0.88)	0.28 (0.10)	-0.02 (0.90)	-0.32 (0.06)	-0.13 (0.45)	-0.22 (0.20)
Group support: conflict at work	0.06 (0.75)	-0.22 (0.20)	-0.22 (0.20)	-0.29 (0.09)	0.09 (0.60)	-0.27 (0.12)	0.04 (0.83)	-0.13 (0.46)	-0.13 (0.44)	-0.28 (0.11)	-0.26 (0.13)	-0.08 (0.65)	-0.06 (0.71)
Noncooperation between groups conflict	-0.07 (0.67)	0.14 (0.44)	0.11 (0.53)	0.14 (0.42)	-0.06 (0.75)	0.14 (0.43)	0.06 (0.72)	0.17 (0.34)	0.07 (0.69)	0.21 (0.23)	0.15 (0.38)	0.05 (0.79)	0.05 (0.79)
Quantitative workload	0.12 (0.51)	0.28 (0.10)	**0.34 (0.04)**	0.25 (0.14)	0.28 (0.11)	0.03 (0.87)	0.22 (0.21)	-0.28 (0.11)	**-0.32 (0.05)**	0.06 (0.71)	-0.21 (0.23)	-0.09 (0.61)	0.04 (0.84)
Resources at work control	0.05 (0.77)	-0.22 (0.21)	-0.19 (0.28)	-0.18 (0.29)	-0.11 (0.53)	-0.03 (0.88)	-0.01 (0.96)	-0.22 (0.21)	0.04 (0.83)	-0.02 (0.91)	-0.27 (0.12)	0.03 (0.87)	-0.07 (0.70)
Responsibility for people	0.15 (0.40)	0.03 (0.85)	-0.09 (0.60)	0.02 (0.90)	-0.02 (0.90)	0.08 (0.64)	0.02 (0.91)	0.17 (0.33)	0.21 (0.21)	0.28 (0.11)	-0.23 (0.18)	0.05 (0.77)	0.01 (0.96)
Role ambiguity	0.17 (0.32)	0.20 (0.26)	0.18 (0.31)	0.22 (0.20)	0.02 (0.89)	0.19 (0.28)	0.10 (0.56)	0.08 (0.64)	-0.28 (0.10)	**0.28 (0.10)**	**0.28 (0.09)**	0.13 (0.48)	**0.26 (0.17)**
Role conflict	0.17 (0.33)	0.30 (0.09)	**0.29 (0.08)**	**0.32 (0.05)**	-0.02 (0.93)	0.22 (0.20)	0.12 (0.50)	-0.02 (0.94)	-0.34 (0.06)	0.21 (0.22)	0.23 (0.19)	0.10 (0.57)	0.14 (0.41)
Skill underutilization	-0.29 (0.10)	-0.09 (0.62)	-0.04 (0.81)	-0.01 (0.99)	-0.09 (0.60)	0.15 (0.39)	-0.17 (0.32)	-0.16 (0.36)	0.17 (0.34)	0.18 (0.31)	-0.21 (0.22)	0.27 (0.12)	0.24 (0.17)
Task control at work	-0.25 (0.15)	-0.17 (0.33)	-0.17 (0.33)	-0.19 (0.28)	-0.03 (0.86)	-0.09 (0.61)	0.02 (0.89)	-0.02 (0.91)	0.25 (0.14)	-0.03 (0.87)	-0.19 (0.27)	0.05 (0.78)	-0.04 (0.85)
Variance in workload	-0.30 (0.09)	0.01 (0.99)	0.10 (0.58)	0.02 (0.92)	0.12 (0.51)	0.002 (0.99)	-0.02 (0.93)	**-0.36 (0.04)**	**-0.36 (0.03)**	0.14 (0.44)	-0.13 (0.45)	0.07 (0.70)	0.12 (0.51)
**Buffer factor**													
Fellow workers	-0.09 (0.60)	-0.06 (0.73)	-0.06 (0.75)	-0.11 (0.54)	0.05 (0.78)	-0.15 (0.40)	0.01 (0.96)	-0.01 (0.94)	-0.14 (0.43)	-0.13 (0.45)	-0.02 (0.92)	-0.24 (0.18)	-0.03 (0.85)
Head nurse	0.02 (0.89)	0.10 (0.57)	0.18 (0.30)	-0.03 (0.88)	0.26 (0.13)	-0.19 (0.29)	-0.02 (0.92)	**-0.32 (0.06)**	**-0.24 (0.16)**	-0.05 (0.78)	-0.23 (0.19)	-0.16 (0.36)	-0.19 (0.27)
Spouse, friends & family	-0.04 (0.81)	0.02 (0.89)	-0.01 (0.94)	-0.04 (0.82)	-0.04 (0.81)	-0.02 (0.90)	**0.33 (0.05)**	0.03 (0.88)	-0.05 (0.78)	-0.04 (0.82)	**-0.44 (0.01)**	-0.13 (0.46)	0.09 (0.59)
**Weekly psychosocial variable [c]**													
CES-D depression score	0.22 (0.20)	0.14 (0.43)	0.15 (0.38)	0.12 (0.49)	0.18 (0.31)	0.001 (0.99)	-0.08 (0.64)	0.07 (0.68)	-0.17 (0.34)	0.19 (0.28)	0.05 (0.79)	-0.03 (0.85)	0.11 (0.53)
POMS anger subscale	0.07 (0.70)	0.07 (0.68)	0.15 (0.40)	0.07 (0.69)	0.13 (0.47)	-0.04 (0.82)	-0.24 (0.16)	-0.13 (0.45)	-0.19 (0.28)	0.15 (0.38)	-0.001 (0.99)	-0.08 (0.64)	-0.06 (0.74)
POMS confusion subscale	0.05 (0.75)	-0.04 (0.82)	0.03 (0.87)	0.03 (0.85)	-0.03 (0.87)	-0.08 (0.67)	-0.31 (0.07)	-0.01 (0.98)	-0.21 (0.21)	0.20 (0.27)	0.14 (0.42)	0.13 (0.48)	0.20 (0.26)
POMS depression subscale	0.08 (0.64)	0.12 (0.50)	0.17 (0.34)	0.10 (0.56)	0.19 (0.27)	-0.03 (0.85)	-0.15 (0.40)	0.02 (0.89)	-0.18 (0.31)	0.23 (0.18)	0.02 (0.90)	-0.06 (0.72)	0.03 (0.86)
POMS frustration subscale	0.13 (0.45)	0.10 (0.57)	0.21 (0.22)	0.13 (0.47)	0.25 (0.14)	-0.07 (0.67)	-0.22 (0.21)	-0.19 (0.28)	-0.19 (0.26)	0.05 (0.79)	**-0.33 (0.05)**	-0.18 (0.32)	-0.18 (0.30)
POMS tension subscale	0.05 (0.80)	0.12 (0.49)	0.18 (0.29)	0.17 (0.32)	0.05 (0.76)	0.15 (0.40)	-0.24 (0.17)	-0.03 (0.87)	-0.08 (0.64)	**0.34 (0.04)**	0.14 (0.43)	0.06 (0.73)	0.05 (0.77)
POMS vigor subscale	0.11 (0.53)	0.22 (0.21)	0.24 (0.16)	0.23 (0.19)	**0.24 (0.17)**	-0.02 (0.91)	-0.11 (0.54)	0.12 (0.49)	-0.23 (0.18)	0.08 (0.66)	0.13 (0.44)	0.01 (0.93)	0.10 (0.56)
Total POMS score	0.12 (0.50)	0.16 (0.36)	0.24 (0.16)	0.19 (0.27)	0.22 (0.21)	0.001 (0.99)	-0.27 (0.12)	-0.02 (0.92)	-0.24 (0.16)	0.21 (0.22)	-0.01 (0.94)	-0.05 (0.80)	0.05 (0.77)

Abbreviation: CES-D, Center for Epidemiologic Studies-Depression; POMS, Profile of Mood Status Short Form; WBC, white blood cells

a. Analyzed using Pearson partial correlation between variables after adjusting for age and smoking status
b. Individual mean values of the variables obtained from NIOSH (National Institute for Occupational Safety and Health) General Job Stress Questionnaire were calculated with the data from 1st month to 9th month
c. Individual mean values were calculated with the data from 6th week to 33rd week

Appendix 3. Results for Hydrocortisol according to the different adjustment strategy

	Hydrocortisol	
	**Adjusted for age and smoking status**	**Adjusted for age, smoking status, and current use of oral contraceptive**
**Monthly psychosocial variables from NIOSH GJSQ [a]**		
**Job stressor**		
Clashes: conflict at work	**-0.48 (0.003)**	**-0.47 (0.01)**
Control & influence at work	**0.59 (0.0002)**	**0.61 (0.0002)**
Decision process control at work	**0.35 (0.04)**	**0.35 (0.04)**
Group support: conflict at work	**0.48 (0.003)**	**0.49 (0.004)**
Noncooperation between groups conflict	-0.15 (0.37)	-0.20 (0.28)
Quantitative workload	-0.19 (0.27)	-0.20 (0.26)
Resources at work control	**0.50 (0.002)**	**0.49 (0.004)**
Responsibility for people	0.22 (0.20)	0.25 (0.16)
Role ambiguity	**-0.52 (0.001)**	**-0.58 (0.001)**
Role conflict	**-0.53 (0.001)**	**-0.56 (0.001)**
Skill underutilization	**0.39 (0.02)**	**0.41 (0.02)**
Task control at work	**0.56 (0.0003)**	**0.58 (0.0004)**
Variance in workload	-0.14 (0.43)	-0.16 (0.36)
**Buffer factor**		
Fellow workers	**0.34 (0.04)**	0.31 (0.07)
Head nurse	**0.36 (0.03)**	**0.33 (0.05)**
Spouse, friends & family	0.28 (0.10)	0.29 (0.11)
**Weekly psychosocial variable [b]**		
CES-D depression score	**-0.42 (0.01)**	**-0.45 (0.01)**
POMS anger subscale	-0.32 (0.06)	**-0.38 (0.03)**
POMS confusion subscale	**-0.33 (0.05)**	**-0.38 (0.03)**
POMS depression subscale	**-0.35 (0.04)**	**-0.37 (0.04)**
POMS frustration subscale	-0.32 (0.06)	**-0.34 (0.05)**
POMS tension subscale	**-0.35 (0.04)**	**-0.40 (0.02)**
POMS vigor subscale	-0.11 (0.52)	-0.17 (0.33)
Total POMS score	**-0.37 (0.03)**	**-0.42 (0.01)**
